# The prognostic impact of myeloid co-mutation burden in TP53-mutated AML/MDS after allogeneic stem cell transplantation: a multicenter retrospective analysis

**DOI:** 10.1007/s00277-026-06895-4

**Published:** 2026-03-07

**Authors:** Yao Sun, Shanshan Qin, Lu Wang, Hai Yi, Li Ding, Bo Cai, Na Liu, Yuhang Li, Jiangwei Hu, Zhuoqing Qiao, Fei Li, Daihong Liu, Liping Dou, Liangding Hu

**Affiliations:** 1 Senior Department of Hematology, Chinese People’s Liberation Army General Hospital, Beijing, China; 2 Department of Hematology, The General Hospital of Western Theater Command, Chengdu, China; 3 Sichuan Clinical Research Center for Hematological Disease, Chengdu, China; 4 Branch of National Clinical Research Center for Hematological Disease, Chengdu, China; 5 Department of Hematology, Air Force Medical University, Air Force Medical Center, PLA, No.30, Fucheng Road, Beijing, 100142 China; 6 Senior Department of Hematology, Chinese People’s Liberation Army General Hospital, 8 East Street, Fengtai District, Beijing, 100071 China

**Keywords:** Acute myeloid leukemia, Myelodysplastic syndromes, TP53 mutation, Allogeneic hematopoietic stem cell transplantation (allo-HSCT), Co-mutation

## Abstract

TP53 mutations are associated with poor prognosis in acute myeloid leukemia (AML) and myelodysplastic syndromes (MDS). Allogeneic hematopoietic stem cell transplantation (allo-HSCT) offers a potential cure, but outcomes are suboptimal and risk stratification remains challenging. This multi-center, retrospective study analyzed 66 patients with TP53-mutated AML/MDS who underwent allo-HSCT. The study endpoints included overall survival (OS), progression-free survival (PFS), and cumulative incidence of relapse (CIR), non-relapse mortality (NRM), and Graft-versus-host disease-free, relapse-free survival (GRFS). After median 1054-day follow-up, 3-year rates for OS, PFS, CIR, NRM, and GRFS were 47.2%, 39.7%, 37.3%, 23%, and 37.4%, respectively. Survival did not differ between AML and MDS. Univariate analysis showed that < 2 somatic myeloid co-mutations predicted inferior OS (3-y OS: 32% vs. 65.9%, *p* = 0.02) and PFS (27.6% vs. 59.1%, *p* = 0.01). Age > 50 years adversely affected PFS, and complex karyotype showed a negative trend. Multivariate analysis found no independent factors, likely due to sample size and collinearity. A combined risk factor analysis revealed that patients with ≥ 1 adverse factor (either co-mutations < 2 or complex karyotype) had significantly worse OS (3-y OS: 72.6% vs. 37.2%, *p* = 0.04) and PFS (3-y PFS: 67.7% vs. 28.9%, *p* = 0.02) compared to those with neither risk factor. In patients with TP53-mutated AML/MDS undergoing allo-HSCT, a low myeloid co-mutation burden (< 2) is strongly associated with poor outcomes. A composite model integrating co-mutation burden with karyotype may assist in post-transplant risk stratification, offering a practical supplementary parameter when TP53 allelic status is uncertain. This finding requires validation in larger prospective studies.

**Supplementary Information:**

The online version contains supplementary material available at 10.1007/s00277-026-06895-4.

## Introduction

TP53 mutations represent a significant molecular aberration in acute myeloid leukemia (AML) and myelodysplastic syndromes (MDS), occurring in approximately 10% of de novo cases and increasing to 25%−30% in therapy-related myeloid neoplasms (MN) [1]. These mutations are strongly associated with complex cytogenetics, treatment resistance, and dismal clinical outcomes, with reported two-year survival rates of less than 15% in TP53-mutated AML/MDS patients [2]. Allogeneic hematopoietic stem cell transplantation (allo-HSCT) remains the only potentially curative treatment modality; however, outcomes remain suboptimal, with a reported 3-year survival rate of approximately 20% and high relapse rates post-transplantation [3, 4].

The newest revisions to the classification systems for myeloid malignancies by both the World Health Organization (WHO) and the International Consensus Classification (ICC) have introduced significant changes in the diagnostic criteria for TP53-mutated MDS and AML, with notable differences in their interpretation of TP53 alleles [5, 6]. The prognostic impact of the allelic state in patients with myeloid neoplasms remains unclear, with conflicting reports comparing bi-allelic (“multi-hit”, MH) TP53 mutations to mono-allelic (“single-hit”, SH) TP53 mutations [7–10]. Consequently, while TP53 mutation allelic status is currently regarded as the most critical prognostic factor for TP53-mutated MN, there is ongoing debate about the criteria for classifying these mutations and their prognostic significance. Furthermore, routine detection of copy-neutral loss of heterozygosity (cn-LOH) in TP53 is challenging, particularly in developing countries like China, hindering accurate assessment of TP53 allelic status.

Given these challenges, our study aimed to evaluate the prognostic factors influencing outcomes in TP53-mutated AML/MDS patients undergoing allo-HSCT, with the goal of providing guidance for prognosis assessment and treatment selection.

## Materials and methods

### Study design and patient selection

A cohort of patients with TP53-mutated (TP53MT) AML and MDS who underwent allo-HSCT was consecutively enrolled from January 2018 to August 2024 across multiple centers, including the Fifth Medical Centre of the Chinese PLA General Hospital, the First Medical Centre of the Chinese PLA General Hospital, the Air Force Characteristic Medical Centre of the Chinese PLA General Hospital, and the Western Theater Command General Hospital. The diagnosis of MDS and AML is based on the fourth edition of the WHO classification.​​ The inclusion criteria for TP53-mutated MDS and AML required confirmation of a TP53 mutation by next-generation sequencing (NGS), using a variant allele frequency (VAF) threshold of ≥ 2% — a cutoff set in light of recent evidence indicating prognostic significance even at VAF levels below 10% [11]. Data was meticulously collected using standardized electronic forms and compiled by an experienced data administrator. The study was approved by the ethics committee of the PLA General Hospital.

### Next-generation sequencing (NGS) panels

Genomic DNA was extracted from bone marrow aspirates obtained from the patients. Subsequent targeted NGS was performed at each participating institution using customized panels that interrogated 46 genes frequently mutated in myeloid malignancies. All detected variants are summarized in Supplement Table 1.

### Measurable residual disease assessment

Given the multicenter, retrospective nature of this study, measurable residual disease (MRD) was assessed by multiparameter flow cytometry (MFC) using institution-specific protocols. Although the specific antibody panels varied, all were designed to detect leukemia-associated immunophenotypes (LAIPs) common in AML and MDS. The results from all centers were harmonized under a common, pre-specified threshold, with MRD negativity defined as < 0.01% phenotypically aberrant cells.

### Conditioning regimens and graft-versus-host disease prophylaxis

All patients received myeloablative conditioning regimens, ​with individualized dosing parameters tailored to patient-specific factors and institutional protocols. Graft-versus-host disease (GVHD) prophylaxis strategies were stratified by donor type and further individualized based on patient-specific characteristics and institutional guidelines.

### Definitions and assessments

Overall survival (OS) was defined as the duration from transplantation to death from any cause or the last follow-up. Progression-free survival (PFS) encompassed the time from transplantation to relapse or death from any cause. Relapse was assessed based on International Working Group criteria [12, 13]. Non-relapse mortality (NRM) and relative risk followed standard criteria [14, 15]. GVHD was defined according to the established criteria [16]. Graft-versus-host disease-free, relapse-free survival (GRFS) was defined as the time from transplantation to the first occurrence of any of the following events: relapse, death from any cause, or the development of grade III–IV acute GVHD or moderate-to-severe chronic GVHD requiring systemic treatment. Patients without any of these events at the last follow-up were censored. Neutrophil recovery was ascertained as the first three consecutive days with an absolute neutrophil count exceeding 0.5 × 10^9/L, and platelet recovery was defined as the first seven days with a non-transfused platelet count surpassing 20 × 10^9/L.

### Statistical analysis

Survival analyses were performed using Kaplan-Meier methodology with log-rank tests for comparisons. Continuous variables were expressed as mean ± standard deviation (SD) for normally distributed data or median (range) for non-normal distributions. To explore independent prognostic factors, variables with a P value < 0.10 in univariate analysis or those deemed clinically essential were considered for inclusion in multivariate Cox proportional hazards regression models. Given the limited number of events, model parsimony was prioritized to avoid overfitting and ensure stability of the estimates. Consequently, final model selection was based on both statistical thresholds and strong clinical/biological rationale. The threshold for statistical significance was set at a two-tailed P value < 0.05. All analyses were performed using SPSS version 25.0 (IBM Corp., Armonk, NY) and GraphPad Prism version 9.2.0 (GraphPad Software, San Diego, CA). A two-tailed p-value < 0.05 was considered statistically significant. In univariate analyses, the cumulative incidence rate (CIR) and NRM was assessed using the Fine-Gray risk model.

## Result

### Patient characteristics

We retrospectively enrolled 66 patients with TP53-mutated AML/MDS, with a specific focus on comparing clinical characteristics between the MDS (*n* = 27) and AML (*n* = 39) groups, as summarized in Table 1. The overall cohort had a median age of 49 years, and 63.6% of patients presented with a complex karyotype. Compared to the MDS group, AML patients were significantly younger (median age 47 years [range 32–73] vs. 53 years [14–60], *P* = 0.038) and had a lower prevalence of complex karyotype (48.7% vs. 85.2%, *P* = 0.002). No significant difference was observed in gender distribution, with males comprising 63.0% of the AML group and 69.2% of the MDS group. We classified patients based on the allele status of TP53 mutations, categorizing them into groups with only one mutation, two mutations, and one mutation combined with 17/17p- or CK without 17/17p-. Due to the lack of VAF values for 13 patients, we did not differentiate patients with a single mutation based on VAF levels. In the patient with one TP53 mutation, the mean VAF is 53.39%, with values of 47.16% in MDS and 59.91% in AML (*p* = 0.05).

**Table 1:** Patient, disease, and transplant characteristics of TP53-mutated AML/MDS cohort

Characteristics	Total (*n* = 66)	MDS (*n* = 27)	AML (*n* = 39)	*P* value
Age, n(range)	49(14–73)	53(32–73)	47(14–60)	0.038
Male, n(%)	44(66.7%)	17(69.2%)	27(63.0%)	0.595
Complex karyotypes, n(%)	42(63.6%)	23(85.2%)	19(48.7%)	0.002
TP53 allele status, n(%)				
1 mut only	22(33.3%)	4(14.8%)	18(46.2%)	
2 mut	9(13.6%)	4(14.8%)	5(12.8%)	
1 mut + with 17/17p-	11(16.7%)	6(22.2%)	5(12.8%)	
1 mut + CK without 17/17p-	24(36.4%)	13(48.1%)	11(28.2%)	
TP53 VAF, mean (range, n)*	53.39%(2.25–95.6%,*n* = 45)	47.16%(2.25–79.94%*n* = 23)	59.91%(27.74–95.6%, *n* = 22)	0.05
Co-mutations, M(P_25_,P_75_)	2(1,3)	1(0,3)	2(1,3)	0.371
t-myeloid malignancies	2	2	0	
Blasts cells prior to transplantation, n(%)				0.125
< 5%	46(69.7%)	16(59.3%)	30(76.9%)	
≥ 5%	20(30.3%)	11(40.7%)	9(23.1%)	
Donor source, n(%)				0.10
HID	42(63.6%)	14(51.9%)	28(71.8%)	
MSD	19(28.8%)	9(33.3%)	10(25.6%)	
MUD	5(7.6%)	4(14.8%)	1(2.6%)	
Stem Cell Source, n(%)				0.365
PB	62(93.9%)	24(88.9%)	38(97.4%)	
PB + BM	4(6.1%)	3(11.1%)	1(2.6%)	
Precondition regimen, n(%)				0.59
Standard	17(25.8%)	6(22.2%)	11(28.2%)	
Modified&	49(74.2%)	21(77.8%)	28(71.8%)	
DAC only	35(53.0%)	18(66.7%)	17(43.6%)	
DAC + VEN	2(3.0%)	0(0.0%)	2(5.1%)	
DAC + RUX	5(7.6%)	1(3.7%)	4(10.3%)	
DAC + RUX+VEN	7(10.6%)	2((7.4%)	5(12.8%)	
GVHD prophylaxis, n(%)				
With ATG	42(63.6%)	21(77.7%)	21(53.8%)	
With PTCy	6(9.1%)	1(3.7%)	5(12.8%)	
Without ATG & PTCy	18(27.3%)	5(18.5%)	13(33.3%)	
Post-transplant prophylaxis, n(%)				0.149
Non	37(56.1%)	18(66.7%)	19(48.7%)	
Yes	29(43.9%)	9(33.3%)	20(51.3%)	
DLI	1(1.5%)	0(0.0%)	1(2.6%)	
Epigenetic therapy	19(28.8%)	4(14.8%)	15(38.5%)	
HMA+XPO1i	5(7.6%)	4(14.8%)	1(2.6%)	
HMA+BCL2i	1(1.5%)	1(3.7%)	0(0.0%)	
FLT3i	3(4.5%)	0(0.0%)	3(7.7%)	

& The use of regimens with additional drugs such as decitabine beyond the classic ones such as Fludarabine, Busulfan, Cyclophosphamide, or Total Body Irradiation

* The calculation of TP53 VAF excluded 9 patients with more than one TP53 mutation and 13 patients with missing VAF values (including 1 patient with two TP53 mutations)

Abbreviations: *AML *acute myeloid leukemia, *MDS *myelodysplastic syndrome, *HID *haploidentical donor, *MSD *matched sibling donor, *MUD *matched unrelated donor, *PB *peripheral blood, *BM *bone marrow, *PB+BM *combined peripheral blood and bone marrow graft, *DAC *decitabine, *VEN *venetoclax, *RUX *ruxolitinib, *GVHD *graft‑versus‑host disease, *ATG *anti‑thymocyte globulin, *PTCy *post‑transplant cyclophosphamide, *DLI *donor lymphocyte infusion, *HMA *hypomethylating agent, *XPO1i *exportin‑1 inhibitor (selinexor), *BCL2i *BCL‑2 inhibitor (venetoclax), *FLT3i *FLT3 inhibitor (sorafenib), *VAF *variant allele frequency, *cnLOH *copy‑neutral loss of heterozygosity, *LOH *loss of heterozygosity, *CK *complex karyotype, TP53 allele status categories: 1 mut only—single TP53 mutation only; 2 mut—two TP53 mutations; 1 mut + with 17/17p‑—single TP53 mutation with chromosome 17/17p deletion; 1 mut + CK without 17/17p‑—single TP53 mutation with complex karyotype but without 17/17p deletion.

The median number of co-occurring mutations was two, with no significant differences between MDS and AML patients. The frequencies of somatic co-mutations in the overall cohort and by disease subtype are detailed in Table 2. In the overall cohort, the most frequent co-mutations were *TET2* (24%), *WT1* (21%), *DNMT3A* (18%), *ASXL1* (15%), and *CEBPA* (12%). The profile differed by subtype: in the AML subgroup, *WT1* (28%), *TET2* (26%), *DNMT3A* (18%), *CEBPA* (18%), and *ASXL1* (15%) were most common; whereas in the MDS subgroup, *TET2* (22%), *DNMT3A* (19%), *ASXL1* (15%), *SRSF2* (7%), and *BCOR/CSF3R* (7% each) predominated. Additionally, 59.3% of patients in the MDS group had a bone marrow blast percentage < 5% before transplantation, compared to 76.9% in the AML group. Moreover, 74.2% of patients received a modified conditioning regimen that included additional drugs such as decitabine, while 43.9% of patients received post-transplant maintenance therapy to prevent relapse.

**Table 2:** Frequency of somatic myeloid co-mutations in TP53-mutated AML/MDS cohort

Gene	Total (*n* = 66)	MDS (*n* = 27)	AML (*n* = 39)
​TET2	16 (24%)	6 (22%)	10 (26%)
​WT1	14 (21%)	3 (11%)	11 (28%)
​DNMT3A	12 (18%)	5 (19%)	7 (18%)
​ASXL1	10 (15%)	4 (15%)	6 (15%)
​CEBPA	8 (12%)	1 (4%)	7 (18%)
​U2AF1	4 (6%)	1 (4%)	3 (8%)
​FLT3	4 (6%)	0	4 (10%)
​SRSF2	3 (5%)	2 (7%)	1 (3%)
​RUNX1	3 (5%)	0	3 (8%)
​IDH2	3 (5%)	0	3 (8%)
​NRAS	3 (5%)	0	3 (8%)
​BCOR	3 (5%)	2 (7%)	1 (3%)
EZH2	3 (3%)	1 (4%)	2 (5%)
BCORL1	2 (3%)	0	2 (5%)
NF1	2 (3%)	0	2 (5%)
GATA2	2 (3%)	1 (4%)	1 (3%)
CSF3R	2 (3%)	2 (7%)	0

Only genes with a total frequency ≥ 2 are listed; low-frequency mutations are not fully shown

Format: Mutation (Frequency/Percentage)

Mutations that appeared only once: PHF6, CUX1, BRAF, KIT, KDM6A, IDH1, NPM1, KMT2A

### Clinical outcomes

**Fig. 1:**
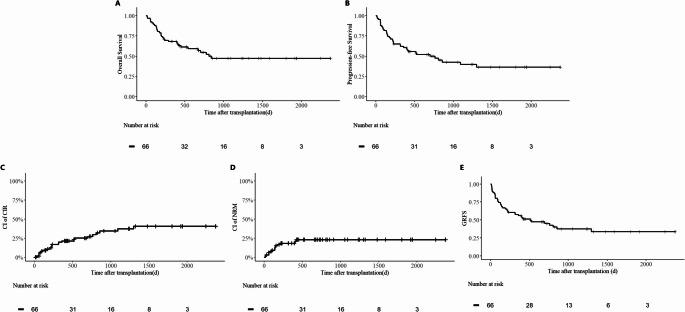
Prognostic of TP53-Mutated AML/MDS Cohort Kaplan-Meier curves show (**A**) overall survival (OS), (**B**) progression-free survival (PFS), (**C**) cumulative incidence of relapse (CIR), (**D**) non-relapse mortality (NRM), and Graft-versus-host disease–free, relapse-free survival (GRFS)

**Fig. 2:**
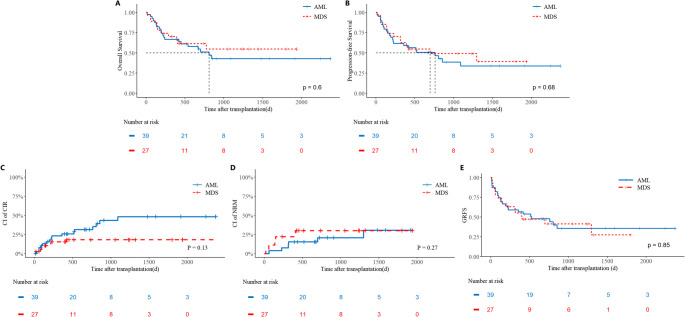
Prognostic Stratification in TP53-Mutated AML/MDS Cohort by diagnosis Comparative outcomes between the acute myeloid leukemia (AML, *n* = 39) and myelodysplastic syndromes (MDS, *n* = 27) subgroups for (**A**) OS, (**B**) PFS, (**C**) CIR, (**D**) NRM, and (**E**) GRFS

The main clinical outcomes are compared in Table 3. Of the 66 patients included in the study, 65 (98.5%) achieved neutrophil engraftment with a median time of 14 days, and 63 (95.5%) achieved platelet engraftment with a median time of 14 days. One patient experienced graft failure of the granulocyte lineage and ultimately succumbed to transplant-related infections. Additionally, three patients experienced primary platelet engraftment failure and died from infections. 27 (40.9%) patients developed grade II-IV aGVHD, and 10 (15.2%) developed grade III-IV aGVHD. cGVHD was observed in 16 patients (23.5%), including 3 cases (4.4%) of extensive cGVHD. After a median follow-up of 1054 days, the entire cohort had 3-year OS, PFS, CIR, NRM, and GRFS rates of 47.2%, 39.7%, 37.3%, 23.0%, and 37.4%, respectively (Fig. 1A-E). Outcomes were comparable between TP53-mutated AML and MDS patients, though a higher CIR was observed in the AML subgroup (3-year CIR: 48.3% vs. 18.1%, *p* = 0.13; Fig. 2A-E).

**Table 3:** Clinical outcomes of TP53-mutated AML/MDS cohort

Characteristics	Total(*n* = 66)	MDS (*n* = 27)	AML (*n* = 39)	*P* value
Implantation[Median(Min-Max)]				
Granulocyte				
Implanted	14(9,24)	14(9,24)	13.5(10,20)	0.72
Not implanted	1(1.5%)	0(0.0%)	1(2.6%)	
Platelet				
Implanted	14(9,31)	14(9,30)	13(9,31)	0.56
Not implanted	3(4.5%)	1(3.7%)	2(5.1%)	
aGVHD				
Ⅱ-Ⅳ	27(40.9%)	10(37.0%)	17(43.6%)	0.59
Ⅲ-Ⅳ	10(15.2%)	4(14.8%)	6(15.4%)	0.95
cGVHD				
Extensive cGVHD	3(4.5%)	2(7.4%)	1(2.6%)	0.36
Limited cGVHD	13(19.7%)	5(18.5%)	8(20.5%)	0.84
3y-NRM	23.0%	30.0%	20.7%	0.27
3y-CIR	37.3%	18.1%	48.3%	0.13
3y-PFS	39.7%	49.3%	33.7%	0.68
3y-OS	47.2%	54.6%	42.9%	0.60
3y-GRFS	37.4%	41.3%	35.5%	0.85

### Univariate analysis

**Fig. 3:**
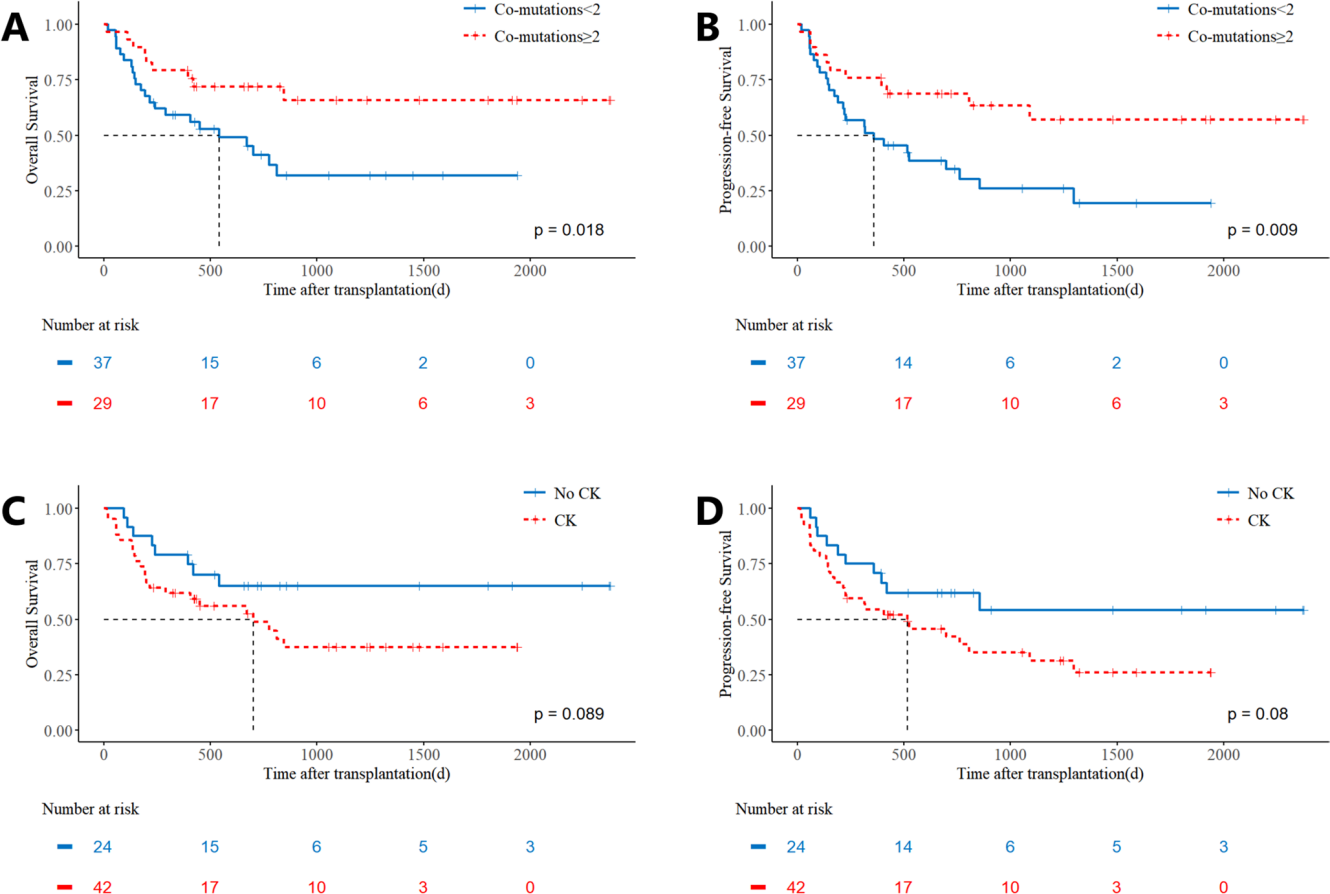
Composite risk stratification based on co-mutation burden and complex karyotype status. Patients were stratified into three groups: no adverse factors (co-mutations ≥ 2 and no complex karyotype), one adverse factor (either co-mutations < 2 or complex karyotype), and two adverse factors (co-mutations < 2 and complex karyotype). Kaplan-Meier curves for (**A**) OS and (**B**) PFS are shown. Patients were grouped by the absence or presence of any adverse factor (one or two). Kaplan-Meier curves for (**C**) OS and (**D**) PFS demonstrate significantly poorer outcomes for patients with any adverse factor

A univariate analysis was conducted to evaluate the impact of various clinical and treatment-related factors on OS and PFS. The factors assessed included the use of modified pretreatment, maintenance therapy, initial diagnosis, complex cytogenetics, age over 50 years, co-mutations < 2 (using this median-based cutoff), and pre-transplantation blast percentage and minimal residual disease (MRD) status (Table 4). The results of the univariate analysis demonstrated that co-mutation < 2 was identified as the only significant adverse prognostic factor for OS (3y-OS: 32% vs. 65.9, *p* = 0.02). Additionally, co-mutations < 2 significantly adversely impacted PFS (3-year PFS: 27.58% vs. 59.06%, *p* = 0.01; Fig. 3A-B), as did age > 50 years (3-year PFS: 31.11% vs. 54.33%, *p* = 0.02; Supplement Fig. 3A-B). Complex karyotype showed a trend toward inferior outcomes (3-y OS: 37.5% vs. 65.1%, *p* = 0.08, 3-y PFS: 31.3% vs. 54.2%, *p* = 0.08). Kaplan-Meier curves for modified pretreatment and maintenance therapy are shown in Supplemental Fig. 1A-F, and for complex cytogenetics in Fig. 3C-D.

**Table 4:** Univariate analysis of factors influencing overall survival and Progression-Free survival in TP53-mutated AML/MDS cohort

Variable	OS (*n* = 66)			PFS (*n* = 66)		
	Odds ratio	95% CI	*P* value	Odds ratio	95% CI	*P* value
Modified Precondition	0.52	0.21–1.26	0.08	0.51	0.23–1.17	0.11
Maintaining	1.05	0.52–2.14	0.88	0.72	0.37–1.38	0.32
AML	1.21	0.59–2.50	0.60	1.15	0.59–2.24	0.68
Complex karyotype	1.98	0.97–4.06	0.09	1.80	0.93–3.47	0.08
Age>50y	1.99	0.96–4.10	0.05	2.25	1.13–4.39	0.02*
Co-mutation <2	2.45	1.21–4.96	0.02*	2.18	1.11–4.27	0.02*
Blasts <5% before HSCT	0.7	0.31–1.58	0.35	0.59	0.28–1.25	0.17
TP53MH*(*n* = 53)	0.91	0.30–2.76	0.87	0.83	0.30–2.32	0.72
FCM-MRD pos before HSCT (46)	1.84	0.53–6.31	0.76	2.06	0.65–6.53	0.22

*TP53 multi-hit (TP53MH) status was defined per International Consensus Classification criteria for all patients with myelodysplastic syndromes and acute myeloid leukemia. A case was defined as TP53MH by the presence of two distinct TP53 mutations (each with a variant allele frequency (VAF) > 10%) or a single TP53 mutation with any of the following: (1) 17p deletion, (2) VAF ≥ 50%, or (3) copy-neutral loss of heterozygosity at the 17p TP53 locus. Definitive classification was not possible for 13 patients due to unavailable VAF data

### Multivariate analysis

To assess whether these factors provided independent prognostic information, a multivariate Cox regression analysis was performed. Based on univariate results and clinical relevance, three variables were included: age (> 50 years), complex karyotype, and co-mutation burden (< 2). For OS, the overall model was significant (Omnibus χ² = 9.824, *P* = 0.020), but none of the individual variables reached statistical independence: co-mutation < 2 (HR = 0.656, 95% CI 0.319–1.348, *P* = 0.251), age > 50 years (HR = 1.878, 95% CI 0.953–3.701, *P* = 0.069), and complex karyotype (HR = 1.585, 95% CI 0.727–3.454, *P* = 0.247). Similarly, for PFS, the overall model showed a trend (Omnibus χ² = 7.799, *P* = 0.050), with no independent variable achieving significance: co-mutation < 2 (HR = 0.681, 95% CI 0.311–1.494, *P* = 0.338), age > 50 years (HR = 1.804, 95% CI 0.859–3.790, *P* = 0.119), and complex karyotype (HR = 1.708, 95% CI 0.723–4.035, *P* = 0.223).

### Combined risk factor analysis

**Fig. 4:**
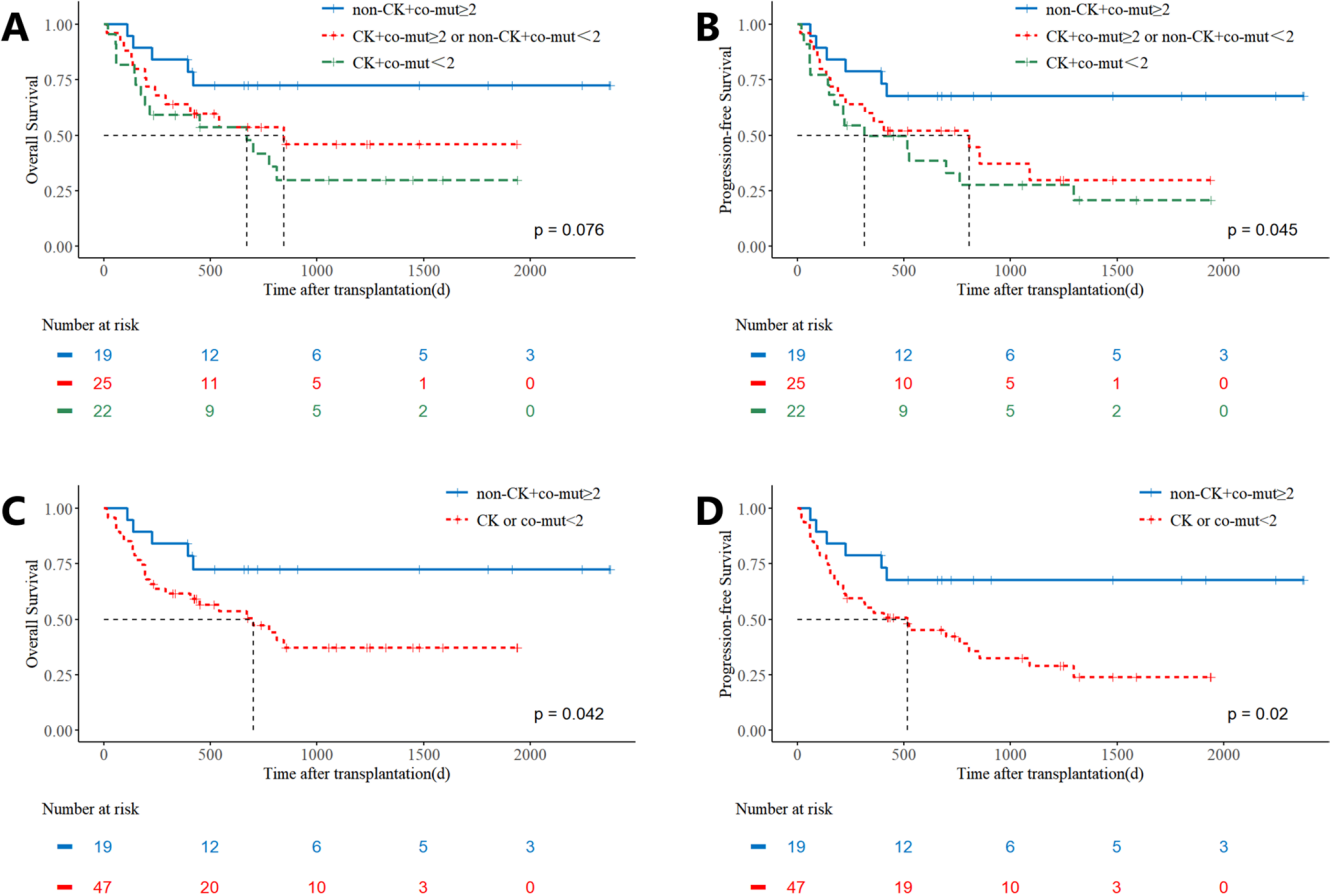
Prognostic Stratification in TP53-Mutated AML/MDS Cohort by genetic prognostic factors. Comparative outcomes stratified by co-mutation burden (low: <2 mutations vs. high: ≥2 mutations) for (**A**) overall survival (OS) and (**B**) progression-free survival (PFS). Outcomes based on the presence or absence of a complex karyotype for (**C**) OS and (**D**) PFS

A strong association was observed between a low myeloid co-mutation burden (< 2) and the presence of complex karyotype (81.5% vs. 51.3%, *p* = 0.01). Although complex karyotype showed only a trend toward inferior prognosis in our univariate analysis, it is a well-established adverse factor in TP53-mutated disease. Given that multivariate analysis did not establish the statistical independence of either factor—likely due in part to their collinearity—yet both hold clinical and biological relevance, we hypothesized that a model combining both might provide more practical risk stratification than either alone. Therefore, we analyzed patients based on the combination of these two factors to assess their composite prognostic utility. Patients were stratified into three groups based on the presence of these adverse factors: those with no adverse factors (co-mutations ≥ 2 and no complex cytogenetics), those with one adverse factor (either co-mutations < 2 or complex cytogenetics), and those with both adverse factors. The results indicated that patients without any adverse factors had the most favorable prognosis, while those with one adverse factor exhibited intermediate outcomes, without significant difference compared to the no-factor group. In contrast, patients with both adverse factors had the worst prognosis, showing statistically significant differences from the no-factor group (Fig. 4A-B). Given the similar survival curves between the one- and two-factor groups, we combined these categories and found that the presence of any adverse factor (one or two) was associated with significantly poorer outcomes compared to the group with no adverse factors ((3-y OS: 72.6% vs. 37.2%, *p* = 0.04, 3-y PFS: 67.7% vs. 28.9%, *p* = 0.02; Fig. 4C-D).

### Subgroup analysis

We conducted an exploratory subgroup analysis to determine whether pre-transplantation blast percentage and MRD status were associated with transplantation outcomes in AML patients. In AML, patients with a pre-transplantation blast percentage < 5% demonstrated significantly better OS and PFS compared to those with a blast percentage ≥ 5% (Supplement Fig. 2A–B). In contrast, among the 30 AML patients in complete remission with available MRD data (8 MRD-positive, 22 MRD-negative), no statistically significant difference in OS, PFS, and CIR was observed between MRD-negative and MRD-positive subjects. (Supplement Fig. 2C–E). These findings highlight the prognostic relevance of low blast burden at transplantation, while the role of pre-transplant MRD in this high-risk TP53-mutated setting remains to be clarified in larger cohorts.

We further performed exploratory analyses of patients receiving different maintenance therapies (Supplement Fig. 3A-B). A total of 19 patients received epigenetic maintenance therapy, including 17 treated with demethylating agents (16 patients with azacitidine and 1 patient with decitabine) and 2 patients receiving a combination of azacitidine and chidamide. The results indicated that there was no significant improvement in OS and PFS for patients receiving epigenetic regulation therapy compared to those without maintenance therapy. One patient who received donor lymphocyte infusion (DLI) and one patient treated with a combination of azacitidine and venetoclax both died approximately one year after transplantation, though this is not reflected in the figure. Encouragingly, 5 patients treated with azacitidine and selinexor remained MRD-negative at a median follow-up of 450 days. Similarly, 3 patients with FLT3-ITD positivity who received sorafenib maintenance therapy post-transplantation remained MRD-negative at a median follow-up of 656 days.

## Discussion

Patients with TP53-mutated AML and MDS undergoing allo-HSCT experience poor outcomes, with current risk stratification relying heavily on TP53 allelic status and complex karyotype—the former often being challenging to determine definitively. In this multicenter, retrospective analysis, we found a strong univariate association between a low myeloid co-mutation burden (<2) and inferior survival outcomes. Although multivariate analysis did not confirm statistical independence from complex karyotype—likely due to sample size constraints—integrating co-mutation burden with karyotype into a composite model effectively stratified patient risk. These findings position myeloid co-mutation burden as a practical and complementary biomarker that may reflect the underlying biological aggressiveness of TP53-mutated disease, particularly when direct allelic interpretation is challenging. The clinical utility of this composite model warrants evaluation in future, larger prospective studies.

The observed association between low co-mutation burden and poor prognosis is supported by both biological and clinical evidence. TP53-mutated myeloid neoplasms typically harbor fewer co-mutations than their TP53-wildtype counterparts, with the mean number increasing stepwise across TP53 allelic subgroups [8, 17–20]. Biologically, this pattern suggests that a low co-mutation burden may indicate the dominance of a single, aggressive TP53-mutant clone, whereas a higher number reflects the coexistence of multiple subclones [21]. Clinically, this model is corroborated by reports showing that the presence of co-mutations is associated with a more favorable prognosis in TP53-mutated MDS-EB or AML [10]. Taken together, fewer myeloid co-mutations may indirectly signal greater clonal dominance and heightened genomic instability, providing a cohesive biological rationale for the prognostic associations observed in our cohort.

Accurate determination of TP53 allelic status, crucial for prognosis, faces persistent technical hurdles. A significant challenge is detecting cn-LOH, which accounts for approximately 12–21% of biallelic cases and requires specialized techniques like SNP arrays or dedicated NGS panels not yet routine in clinical practice [22]. Furthermore, while a high VAF is linked to worse outcomes, the lack of a standardized VAF cutoff (ranging from 10% to 50% across studies) complicates its application [20, 23–26]. This ambiguity is highlighted by findings that AML patients with TP53 VAF ≥ 2% and < 10%—excluded from the “AML with mutated TP53” subtype under ICC criteria—can still have very poor prognosis when concurrent with other high-risk features like complex karyotype [11]. Therefore, in line with recommendations to comprehensively assess TP53-mutated disease, our study pragmatically included all patients with a detectable mutation (VAF ≥ 2%) to capture the full prognostic spectrum of this genetically defined cohort [22]. These persistent limitations underscore why TP53 allelic classification and prognostication remain areas of active refinement, driving the search for supplementary biomarkers that are both informative and clinically accessible. For instance, p53 protein expression has been shown to correlate with mutation and copy number status, offering a potential complementary assay for identifying high-risk patients [27]. In this context, our evaluation of myeloid co-mutation burden aligns with this translational goal, seeking a practical molecular metric that can aid risk stratification when definitive allelic assignment is uncertain.

Regarding therapeutic approaches, while an earlier pilot study suggested potential benefit from decitabine-based preconditioning, our current analysis found no significant prognostic improvement with such regimens, nor with standard azacitidine maintenance—consistent with prior reports [28–30]. However, exploratory data from novel strategies are promising: the exportin-1 inhibitor selinexor (which promotes p53 nuclear retention) combined with azacitidine sustained MRD negativity in five patients, and sorafenib maintenance achieved sustained remission in three with TP53/FLT3-ITD co-mutations [31]. These preliminary findings warrant validation in dedicated trials. Furthermore, in our exploratory analysis with limited sample size, we did not observe a statistically significant association between pre-transplant MRD status and survival outcomes. This finding may be influenced by the small number of events and the uniformly high-risk nature of the cohort, underscoring the need for its evaluation in larger studies. When comparing our study and several reports from China with Western cohorts, the improved 3-year OS (30–60% vs. 20–30%) may be attributed to several factors, including younger median age in our cohort and potential inclusion of patients with isolated chromosome 17 deletions—a subgroup historically associated with more favorable prognosis [4, 10, 20, 32–38]. These differences highlight the importance of considering population characteristics when interpreting transplant outcomes.

Our study has several important limitations that must be considered when interpreting the findings. First, the retrospective design and modest sample size (*n* = 66) inherently limit statistical power and preclude meaningful subgroup analyses. This constraint increases the risk of confounding and means that some of our observations, including the lack of independent significance in multivariate analysis, should be interpreted with caution—they may reflect limited power rather than a true absence of effect. Second, due to the limited sample size, we were unable to perform meaningful analyses on the impact of specific strong leukemic driver mutations (e.g., RUNX1, FLT3) in combination with TP53 mutation—an important area for future investigation with larger cohorts. Third, accurate determination of TP53 allelic status, including cn-LOH detection, was not routinely available, restricting precise classification per contemporary criteria (WHO5/ICC). Fourth, heterogeneity in conditioning and maintenance therapies may have influenced outcomes in unmeasured ways. Fifth, and importantly, our findings are derived from a single retrospective cohort without an external validation set, which limits the generalizability and definitive strength of the conclusions. Finally, the predominantly single-country cohort may limit generalizability to other populations. Consequently, our findings should be viewed as hypothesis-generating, emphasizing the need for validation in larger, prospective, and molecularly well-characterized cohorts where TP53 allelic status is definitively assigned.

This study suggests that a low myeloid co-mutation burden (< 2) in TP53-mutated AML/MDS patients undergoing allo-HSCT may be associated with poorer outcomes. When combined with complex karyotype, it could aid in post-transplant risk stratification. This exploratory finding positions co-mutation burden as a potential supplementary parameter, especially when TP53 allelic status is indeterminate. Prospective validation in larger cohorts is warranted.

## Supplementary Information

Below is the link to the electronic supplementary material.

Supplementary Material 1 (DOCX 534 KB)

## Data Availability

The datasets generated and analyzed during this study are not publicly available to protect patient privacy. The corresponding author will consider requests for data access from qualified researchers on a case-by-case basis.
