# Supplementary material for: The prognostic impact of myeloid co-mutation burden in TP53-mutated AML/MDS after allogeneic stem cell transplantation: a multicenter retrospective analysis
Source: Ann Hematol. 2026 Mar 7;105(4):168. doi: 10.1007/s00277-026-06895-4 (PMC12967432; doi:10.1007/s00277-026-06895-4)
Supplement: Supplementary file 1 — Supplementary Material 1 (DOCX 534 KB) [file 277_2026_6895_MOESM1_ESM.docx]

**Supplementary Material**

**Supplement Figure 1. Univariate analysis of treatment factors and age on survival outcomes.**

Kaplan-Meier curves for (A) overall survival (OS) and (B) progression-free survival (PFS) stratified by age (≤50 years vs. >50 years).


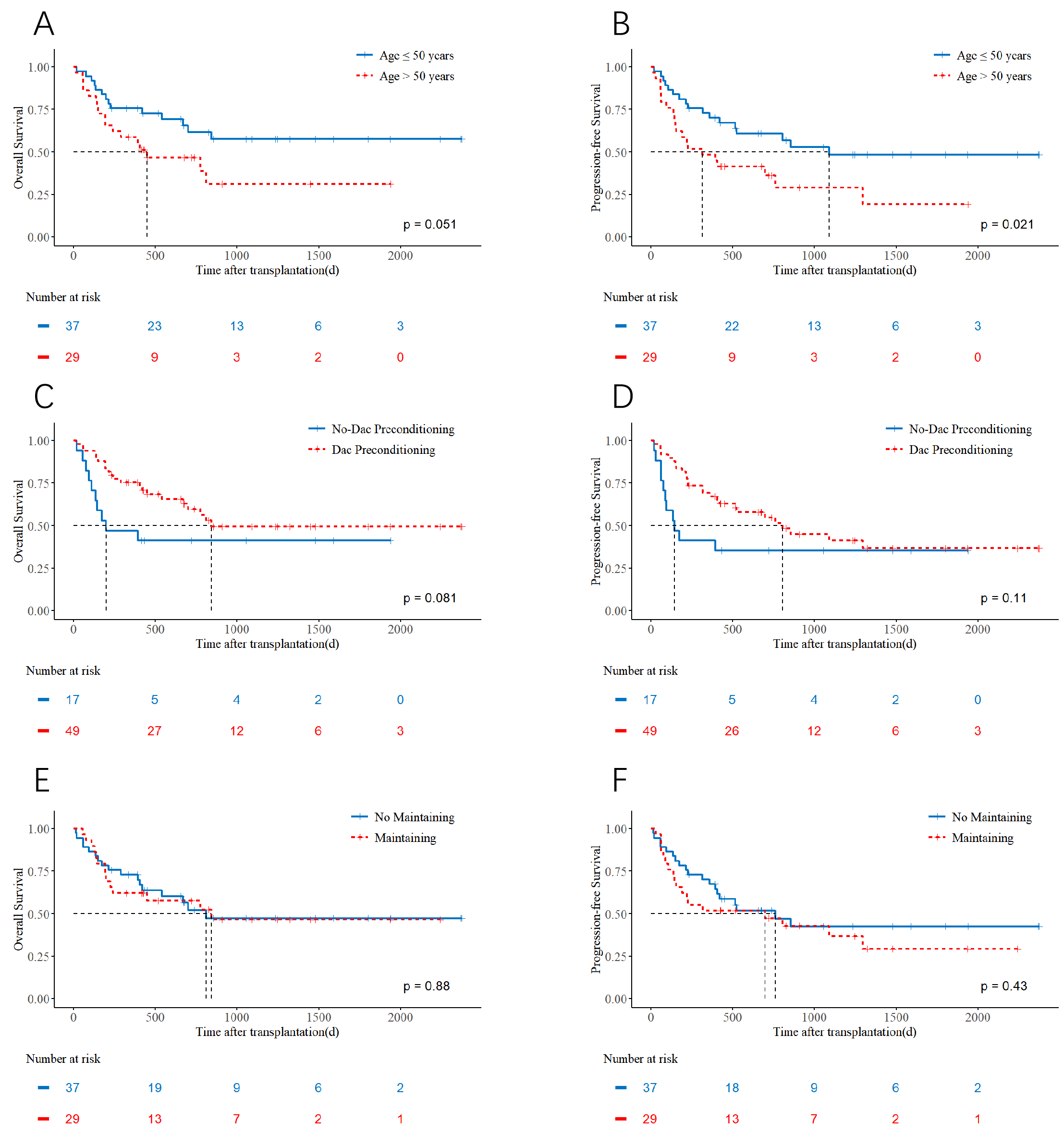


**Supplemental Figure 2. Pre-transplantation blast percentage and MRD status as prognostic factors in TP53-mutated AML.​**​
(A) Overall survival (OS) and (B) progression-free survival (PFS) stratified by pre-transplantation blast percentage (<5% vs. ≥5%). (C) OS, (D) PFS and (E) CIR stratified by pre-transplantation flow cytometry-based minimal residual disease (MRD) status (negative vs. positive).


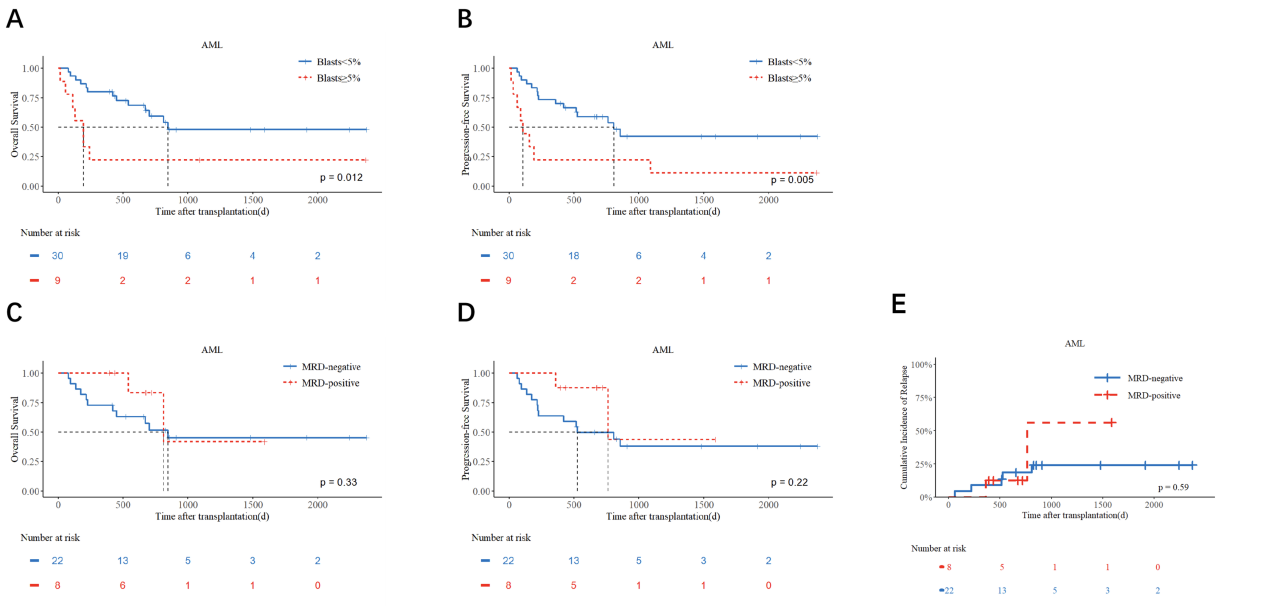


**Supplemental Figure 3. Impact of post-transplantation epigenetic maintenance therapy on survival outcomes.​​**Kaplan-Meier curves comparing (A) overall survival (OS) and (B) progression-free survival (PFS) between patients who received different kinds of maintenance therapy and those who did not receive any maintenance therapy.
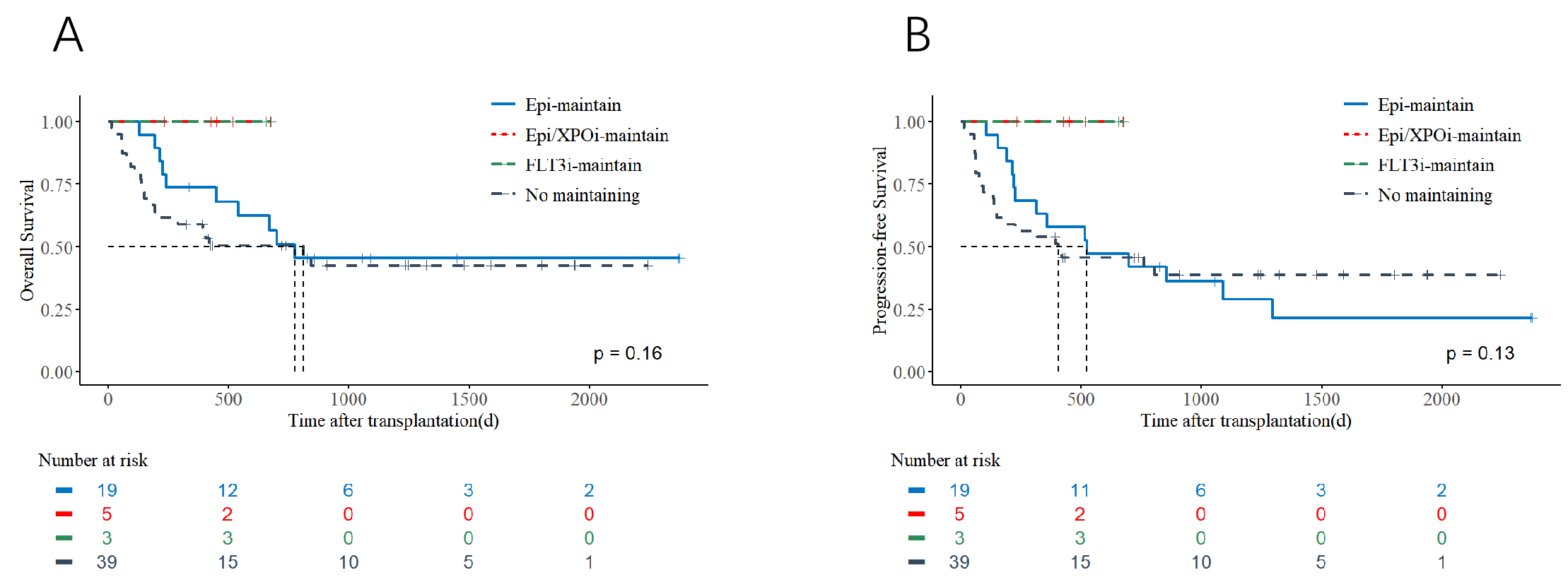


**Supplement Table 1. List of the 46 genes included in our targeted sequencing panel.**

| ***ABL1*** | ***ASXL1*** | ***BCOR*** | ***BCORL1*** | ***BRAF*** |
| --- | --- | --- | --- | --- |
| ***CALR*** | ***CBL*** | ***CEBPA*** | ***CHEK2*** | ***CSF3R*** |
| ***CUX1*** | ***DNMT3A*** | ***EP300*** | ***ETV6*** | ***EZH2*** |
| ***FLT3*** | ***GATA1*** | ***GATA2*** | ***IDH1*** | ***IDH2*** |
| ***JAK3*** | ***KDM6A*** | ***KIT*** | ***KMT2D*** | ***KRAS*** |
| ***JAK2*** | ***MPL*** | ***NF1*** | ***NOTCH1*** | ***NPM1*** |
| ***PHF6*** | ***PTPN11*** | ***RAD21*** | ***RUNX1*** | ***SETBP1*** |
| ***NRAS*** | ***SF3B1*** | ***SMC1A*** | ***SRSF2*** | ***STAG2*** |
| ***STAT3*** | ***SUZ12*** | ***TET2*** | ***U2AF1*** | ***WT1*** |
| ***ZRSR2*** |  |  |  |  |
